# The aftermath of COVID-19: generalized anxiety disorder and burnout among radiology practitioners and interns in Saudi Arabia

**DOI:** 10.3389/fpsyt.2024.1401213

**Published:** 2024-10-09

**Authors:** Khalid M. Alshamrani, Abdulkader A. Alkenawi, Hebah A. Falatah, Waad Alsulami, Faisal A. Alzahrani, Tariq M. Nayta, Abdulrahman H. Alharbi, Mohannad A. Alzahrani, Rahaf H. Almutairi, Bander S. Alshomrani, Sameer E. Tasslaq, Ali M. Aldhebaib

**Affiliations:** ^1^ College of Applied Medical Sciences, King Saud bin Abdulaziz University for Health Sciences, Jeddah, Saudi Arabia; ^2^ King Abdullah International Medical Research Center, Jeddah, Saudi Arabia; ^3^ Ministry of the National Guard - Health Affairs, Jeddah, Saudi Arabia; ^4^ King Saud Medical City, Riyadh, Saudi Arabia; ^5^ King Abdulaziz Hospital and Oncology Center, Jeddah, Saudi Arabia; ^6^ College of Applied Medical Sciences, King Saud bin Abdulaziz University for Health Sciences, Al-Ahsa, Saudi Arabia; ^7^ College of Applied Medical Sciences, King Saud bin Abdulaziz University for Health Sciences, Riyadh, Saudi Arabia

**Keywords:** anxiety disorders, burnout syndrome, COVID-19 pandemic, psychiatry, Kingdom of Saudi Arabia, healthcare workers, radiologic technology

## Abstract

**Background:**

The coronavirus disease (COVID-19) pandemic has presented unprecedented stressors and difficulties for healthcare professionals. This study explored the prevalence of generalized anxiety disorders and burnout among radiology practitioners and interns in various hospitals in Saudi Arabia after the end of the COVID-19 global public health emergency.

**Methods:**

A cross-sectional survey of 230 radiology practitioners and interns was conducted between October and November 2023. This study utilized the Generalized Anxiety Disorder 7-item (GAD-7) scale and Maslach Burnout Inventory-Human Services Survey for Medical Personnel (MBI-HSS-MP) 22-item questionnaire, employing a non-probability convenience sampling method. The average scores of the individual components constituting the GAD-7 scale and each burnout scale were calculated, and statistical analyses were conducted using the Mann-Whitney U and Kruskal-Wallis H nonparametric tests.

**Results:**

Of 382 radiology practitioners and interns, 230 (60.2%) responded to the survey. Notably, 42.6% of the participants reported experiencing GAD. Regarding burnout, 82.3% were at moderate-to-high risk for emotional exhaustion, 93.5% for depersonalization, and 52.1% for personal achievement. The 31–40 years age group showed significantly higher burnout rates (p = 0.001) compared with the other age groups. Those with more than three years of experience had notably higher emotional exhaustion scores (p = 0.002) and a nearly significant increase in depersonalization scores (p = 0.051) than those with less experience.

**Discussion:**

Our study revealed that 42.6% of radiology practitioners and interns experienced GAD, with the majority facing significant burnout. Furthermore, our research indicates a decline in GAD levels among radiology practitioners and interns compared with the peak COVID-19 pandemic period. It also showed a significant increase in both the incidence and severity of burnout, surpassing pre-pandemic levels in a comparable cohort. These findings emphasize the pressing challenges of GAD and burnout among healthcare workers, especially radiology professionals.

## 1 Introduction

Healthcare workers (HCWs), including doctors, nurses, and allied health professionals, are at the forefront of care provision, often under high-stress conditions. This stress, compounded by long working hours, high expectations, and the emotional toll of patient care, can significantly elevate the risk of developing burnout and anxiety disorders, including Generalized Anxiety Disorder (GAD) (1). The COVID-19 pandemic has intensified stress, thereby increasing the risk of burnout and GAD (2–4).

Stress, a concept with diverse interpretations in the literature, can generally be defined as a response to the perceived threat of something valued (5). Before the COVID-19 pandemic, more than 60% of HCWs, including physicians, advanced care providers, and nurses, reported high levels of stress (6, 7). During the pandemic, stress levels surged further. A 2022 survey by the American Nurses Foundation, involving 11,964 nurses, revealed that over 70% had recently experienced stress (8). Additionally, nearly 30% of the 2,373 surveyed physicians reported high stress levels (6). Another study found that 57% of HCWs experienced acute stress during the early stages of the pandemic (2). In the United States, the Web-based Implementation of the Science for Enhancing Resilience (WISER) study, which involved 2,310 HCWs identified three main types of stressors: work-related (49%), including job demands and work relationships; personal life (32%), such as family issues; and blended stressors (19%), such as financial concerns and work-life balance (1).

Prolonged periods of elevated stress levels are precursors to burnout (9), a term coined by Freudenberger (1974) (10). Burnout is characterized by decreased motivation, emotional depletion, and cynicism, and manifests as mental and emotional exhaustion, depersonalization, and a diminished sense of professional achievement (11). In recent years, research on burnout has surged due to its detrimental effects on HCWs. During the pandemic, emotional exhaustion among nurses increased from 40.6% in 2019 to 49.2% between 2021 and 2022 (12). Similar trends were observed among physicians and other HCWs (12). Stress and burnout among HCWs not only affect their well-being but also have detrimental effects on patient care. A correlation exists between nurses’ stress levels (13) and physician burnout (14) with the increase in medical errors. Furthermore, hospital-acquired infections were associated with burnout among HCWs, highlighting their impact on patient safety (15). Lower patient satisfaction has been observed (16) in environments where burnout is prevalent. Importantly, research has indicated that in settings with high levels of nurse burnout, patients tend to have longer hospital stays and a higher risk of mortality (17). The incidence of burnout in radiology is increasing globally, with overall and high/severe burnout rates at 88% and 62%, respectively (18). Increasing workloads, high-stress environments, and evolving imaging technologies have significantly contributed. Radiologists endure long hours, high diagnostic expectations, and limited patient interactions, making them particularly vulnerable (18, 19). A 2020 survey by the American College of Radiology (ACR) found that nearly one-third of radiologists rated their burnout as severe, citing workload, administrative burden, and pressure to maintain productivity as the primary factors (20).

Research has indicated a significant link between increased stress levels and the onset of anxiety and depression (6). GAD, characterized by excessive and persistent worry, affects up to 20% of adults (21–23). The prevalence of GAD among HCWs is determined by a multifaceted combination of work-related, personal, and sociocultural factors. Potent stressors include high-stress job environments, increased workloads, shortage of Personal Protective Equipment (PPE), negative media coverage, insufficient support from authorities, exposure to human suffering, and heightened risk of COVID-19 infection (24–27). In 2020, twenty research studies conducted in eight different countries examined the prevalence of GAD among HCWs during the COVID-19 pandemic. Utilizing the GAD 7-item (GAD-7) anxiety scale as a measurement tool, these studies found prevalence rates varying between 12% and 48%, with an average of 30.5% across the board (2, 24, 28–37). HCWs employed in high-stress areas, such as emergency departments, intensive care units (ICU), and infectious disease wards, face an elevated risk of psychiatric issues (27). Those in direct patient care roles exhibited higher levels of fear, depression, and anxiety than administrative personnel because of their direct exposure to COVID-19 patients (38). A recent study found that a significant proportion of radiology staff reported anxiety and burnout, although at lower rates than those in the broader hospital cohort (39).

Numerous studies in Saudi Arabia (KSA) have identified GAD and burnout among HCWs, including doctors, residents, nurses, radiographers, and physiotherapists (40–60). and undergraduate health sciences students in fields such as medicine, dentistry, rehabilitation sciences, radiological sciences, nursing, and pharmacy, both before and during the COVID-19 pandemic (60–73). However, the specific effects of GAD and burnout on radiology practitioners and interns following the World Health Organization’s (WHO) declaration on May 5, 2023 (74), which marked the end of the global Public Health Emergency (PHE) for COVID-19, are yet to be determined. This is due to the current lack of extensive research focusing on these impacts in this region and among radiology professionals. Our research aimed to address this gap by examining the prevalence of GAD and burnout among radiology practitioners, including technicians, technologists, specialists, senior specialists, consultants, and interns. We also investigated how demographic factors influenced the prevalence of GAD and burnout in these groups.

In Saudi Arabia, a Radiology Technologist or Specialist is defined as a professional with a bachelor’s degree, which includes four years of undergraduate education and a one-year internship. A Radiology Technician is recognized as a professional who has achieved a diploma after two years of undergraduate study. A Senior Specialist is a professional who has completed either a master’s degree followed by two years of experience or a four-year residency training program in Radiology Technology and Medical Imaging. A consultant in this field is a professional who holds a PhD or Doctor of Philosophy degree (75–77).

## 2 Material and methods

### 2.1 Participants and procedure

A descriptive cross-sectional questionnaire study was conducted between October and November 2023 among radiology practitioners and interns working in the medical imaging departments of several tertiary hospitals in Saudi Arabia, including King Abdulaziz Medical Cities (KAMCs) in Jeddah, Riyadh, Al Ahsa, King Saud Medical City (KSMC) in Riyadh, and King Abdulaziz Hospital and Oncology Center (KAHOC) in Jeddah. The KAMCs are part of the Ministry of National Guard Health Affairs (MNG-HA), a government-funded health system established in 1983. KAMC consists of medical cities located in various regions, including Riyadh, Jeddah, and Al Ahsa. These medical cities are considered to have some of the most comprehensive healthcare facilities in Saudi Arabia. KSMC is a tertiary Ministry of Health hospital that began serving the people of Riyadh in 1956. It currently has a capacity of 1,400 beds and employs over 8,000 staff. Medical City includes major hospitals, such as the General Hospital, Pediatrics and Maternity Hospitals, Dental Center, and King Fahad Charity Kidney Center. KAHOC, one of the most modern hospitals affiliated with the Ministry of Health, is located in southern Jeddah. Built in 1990 and designed according to the latest international standards, the hospital spans more than 200,000 square meters and contains 445 beds across various specialties. We invited the entire population of 382 radiology practitioners and interns to participate in the study. Using the Raosoft^®^ Sample Size Calculator (Raosoft inc.) (78), we determined that the recommended sample size was 192, based on a 5% margin of error and a 95% confidence level. This study included radiology practitioners (technicians, technologists, specialists, senior specialists, and consultants) and interns. Radiologists and nurses were excluded from the study. Participants were approached through non-probability convenience sampling via email and WhatsApp invitations. The questionnaire was conducted online through an electronic survey tool (i.e., Google Form).

### 2.2 Study measures

Radiology practitioners and interns were asked to complete two well-established, previously used, and validated assessment tools: the Generalized Anxiety Disorder 7-item (GAD-7) scale questionnaire (79) and the Maslach Burnout Inventory-Human Services Survey for Medical Personnel (MBI-HSS-MP) (80). The questionnaires were reviewed by four senior radiology practitioners with extensive experience in radiological technology. This review ensured that the questionnaires were specifically targeted towards our study demographics, clear and concise, and maintained a focused and purposeful approach.

The GAD-7 is a 7-item anxiety scale tool with robust criterion validity for identifying potential GAD cases. It also serves as an excellent measure of severity, with higher scores indicating greater functional impairment and more days of disability. A score of 10 or higher on the GAD-7 is considered a suitable threshold for detecting potential cases of GAD. Scores of 5, 10, and 15 indicate mild, moderate, and severe levels of anxiety on the GAD-7, respectively. For the purpose of this study, a cutoff GAD-7 score of 8 or higher indicates the presence of anxiety symptoms. Each item was assessed on a 7-point frequency rating scale, ranging from 0 (never) to 6 (every day). The subscale scores were calculated and interpreted separately for each respondent. The GAD-7 scale demonstrated good internal consistency and reliability, with a Cronbach’s alpha of 0.868.

The MBI-HSS-MP consists of 22 items designed to measure burnout among medical personnel. It encompasses three distinct dimensions/subscales:

1. Emotional Exhaustion (EE) (9-items): This dimension evaluates emotional responses stemming from excessive work pressure, feelings of being physically and emotionally drained, and decreased enthusiasm for work. The EE score encompasses nine items, with a score range of 0–54. Scores below 19 indicate low burnout, scores between 19 and 26 signify moderate burnout, and scores exceeding 26 indicate high burnout. The EE subscale demonstrated high internal consistency reliability with a Cronbach’s alpha of 0.889.
2. Depersonalization (DP) (5-items): This dimension assesses attitudes and feelings toward work, including cynicism, callousness, and impersonal responses toward patient care, as well as reduced empathy and increased cynicism. DP was assessed using five items with a score range of 0–30 points. Scores below 6 denote low burnout, scores ranging from 6 to 9 indicate moderate burnout, and scores surpassing 9 indicate high burnout. The DP subscale demonstrated high internal consistency reliability with a Cronbach’s alpha of 0.791.
3. Personal Accomplishment (PA) (8-items): This dimension measures feelings of competence, successful achievement, and the meaningfulness of one’s work. The PA evaluation comprised eight items with a score range of 0–48 points. Scores exceeding 39 indicate low burnout, scores between 34 and 39 indicate moderate burnout, and scores below 34 reflect high burnout (11, 80, 81). The PA subscale demonstrated high internal consistency reliability with a Cronbach’s alpha of 0.887.

The scoring of the subscales employed two methods: summation (SUM) and average (AVE), as outlined by Maslach et al. (2018) (80). These methods were selected to facilitate comparison with recent publications. In both methods, higher scores on the EE and DP scales indicated greater levels of burnout, whereas lower scores on the PA scale signified higher degrees of burnout. The scores for each burnout dimension were derived by calculating the arithmetic mean of the individual items within each burnout scale (11, 81–84).

### 2.3 Ethical consideration

The local Institutional Review Board (IRB) approved this research under protocol number SP22J/100/08. Participation in the study was voluntary, and the participants provided written informed consent before completing the questionnaire. Written informed consent was obtained by including a consent form at the beginning of the Google Form questionnaire. Participants were required to read the consent form carefully and indicate their agreement by selecting an option before proceeding to the rest of the questionnaire. All responses were anonymous and confidential, and we followed the principles of the Declaration of Helsinki throughout the study. The electronic survey application created a password-protected Microsoft Excel file without identifying participant information.

### 2.4 Statistical analyses

The statistical analyses were conducted in four phases. The first phase involved a descriptive analysis that provided demographic details (i.e., counts and percentages) along with calculating the sample’s mean and standard deviation (SD) for the scores. In the second phase, Cronbach’s alpha test was applied to evaluate the internal consistency of the EE, DP, and PA subscales. The third phase utilized the Shapiro-Wilk test to assess the normality of the data distribution (i.e., scores). The fourth and final phases involved conducting Mann-Whitney U and Kruskal-Wallis H nonparametric tests, accompanied by Dunn’s *post-hoc* test, to explore potential differences in score means across the study groups. All analyses were performed using SPSS version 23, with the threshold for statistical significance set at p < 0.05.

## 3 Results

### 3.1 Characteristics of the participants

**Table 1** presents the sociodemographic characteristics of participants. Of the 382 radiology practitioners and interns contacted, 230 participated, resulting in a response rate of 60.2%. The sex distribution was 66.5% male and 33.5% female. The mean age of the participants was 31.7 years (SD = 8.2) and 53% were aged between 21-30 years. The majority were Saudi nationals (97.8%) and had earned their degrees in Saudi Arabia (90.4%). Approximately half of the respondents were single. A significant proportion (42.6%) specialized in radiography, mammography, and fluoroscopy, and 63.9% were categorized as technologists or specialists.

**Table 1:** Sociodemographic characteristics of the participants.

Variable		Total Sample (n = 230)	
		n	%
**Gender**	Male	153	66.5
	Female	77	33.5
**Age**	21 - 30	122	53.1
	31 - 40	64	27.8
	41 - 60	44	19.1
**Nationality**	Saudi Nationality	225	97.8
	Other Nationality	5	2.2
**Country of your Radiologic Technology degree**	Saudi Arabia	208	90.4
	Non-Saudi	22	9.6
**Marital Status**	Single	120	52.2
	Married	102	44.3
	Divorced	8	3.5
**Healthcare Institution**	KAMC-Jeddah	72	31.3
	KAMC-Riyadh	22	9.6
	KAMC-Al Ahsa	40	17.4
	KAHOC	15	6.5
	KSMC	81	35.2
**Medical Imaging Division/Subspeciality**	Radiography/Mammography/Fluoroscopy	98	42.6
	Magnetic Resonance Imaging (MRI)	22	9.6
	Computed Tomography (CT)	49	21.3
	Nuclear Medicine	16	7
	Ultrasonography	25	10.9
	Angiography	20	8.7
**Professional Rank**	Technician	20	8.7
	Technologist/Specialist	147	63.9
	Senior Specialist	36	15.7
	Consultant	4	1.7
	Intern	23	10
**Years of Experience**	Less than 1 year	55	23.9
	1-3 years	35	15.2
	4 - 5 years	36	15.7
	6 + years	104	45.2

- $\text{Percentage of Responses }\left(\%\right)=\frac{Number\;of\;Responses\;\left(n\right)}{230}\times 100$ - KAMC, King Abdulaziz Medical City.

- KSMC, King Saud Medical City.

- KAHOC, King Abdulaziz Hospital and Oncology Center.

### 3.2 Prevalence of anxiety symptoms among radiology practitioners and interns

**Table 2** presents the participants’ responses to the GAD-7. The data indicated that a significant portion of participants reported experiencing symptoms on several days, more than half of the days, or nearly every day. Specifically, 79.6% felt nervous, anxious, or on edge; 67% found it challenging to stop or control worrying; 74.8% were excessively concerned about various things; 77.8% had difficulty relaxing; 59.1% felt too restless to remain still; 76.5% became easily annoyed or irritable; and 64.8% felt fearful as though something terrible might occur.

**Table 2:** Prevalence of anxiety symptoms among radiology practitioners and interns.

GAD-7 Items							Not at all		Several days		More than half the days		Nearly everyday	
							n	%	n	%	n	%	n	%
1						Feeling nervous, anxious, or on edge	47	20.4	131	57	41	17.8	11	4.8
2						Not being able to stop or control worrying	76	33	108	47	32	13.9	14	6.1
3						Worrying too much about different things	58	25.2	111	48.3	44	19.1	17	7.4
4						Trouble relaxing	51	22.2	119	51.7	43	18.7	17	7.4
5						Being so restless that it’s hard to sit still	94	40.9	81	35.2	45	19.6	10	4.3
6						Becoming easily annoyed or irritable	54	23.5	122	53	41	17.8	13	5.7
7						Feeling afraid as if something awful might happen	81	35.2	105	45.7	26	11.3	18	7.8

$\text{Percentage of Responses }\left(\%\right)=\frac{Number\;of\;Responses\;\left(n\right)}{230}\times 100$ .

### 3.3 Variations in mean GAD-7 scores, stratified by study variables

**Figure 1:**
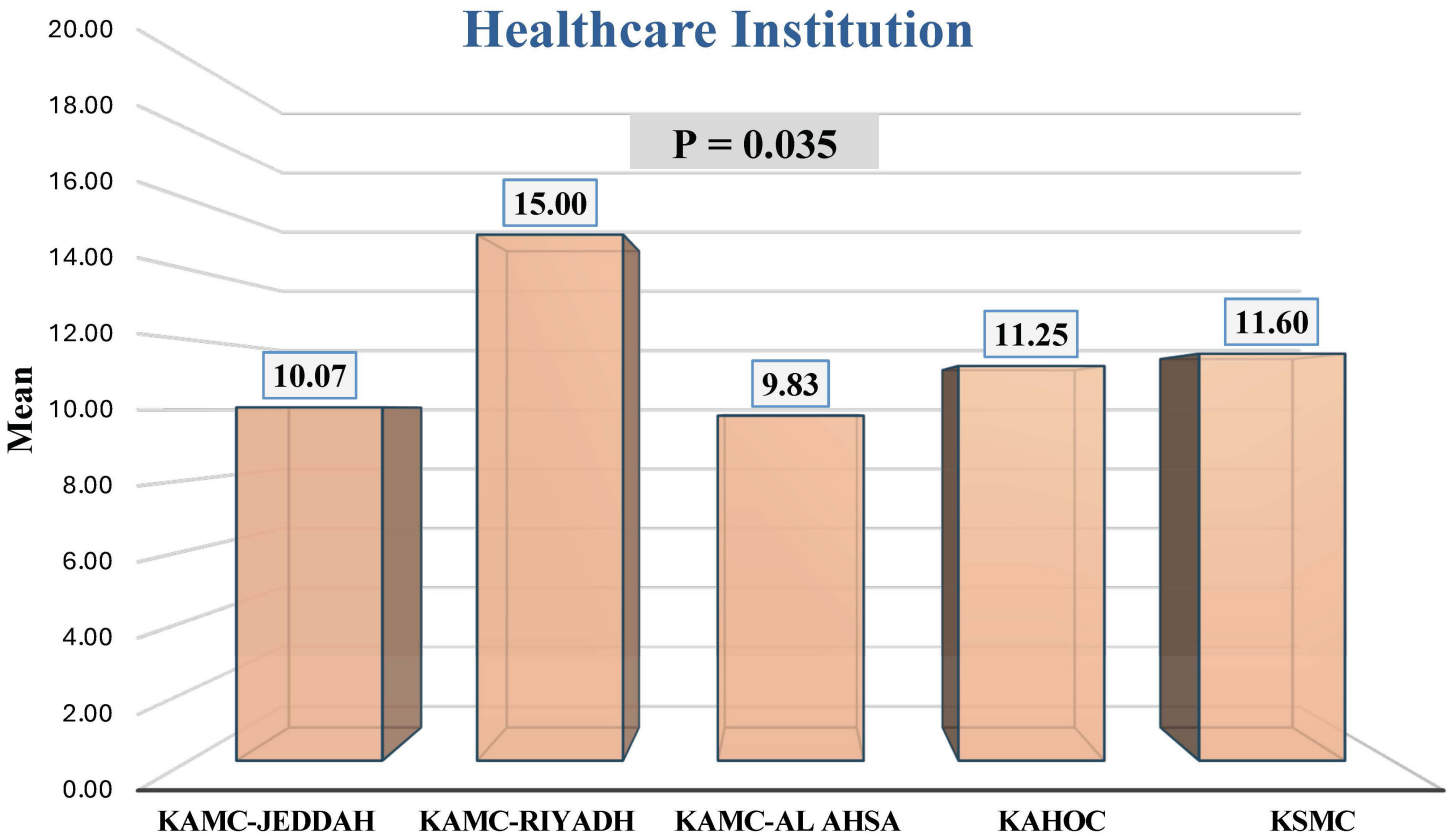
Comparison of mean GAD-7 scores for participants with anxiety symptoms, categorized by healthcare institution.

**Table 3** presents the differences in mean GAD-7 scores for participants with anxiety symptoms (i.e., GAD-7 ≥ 8), categorized by study variables. In the total sample, 42.6% reported experiencing GAD. Variations in mean GAD-7 scores among radiology practitioners and interns with anxiety were linked to the institution with which they were affiliated (**Figure 1**). Individuals with GAD affiliated with KAMC-Riyadh reported higher mean anxiety scores than those affiliated with KAMC-Jeddah (p = 0.015), KAMC-Al Ahsa (p = 0.001), and KAHOC (p = 0.034). The mean GAD-7 scores of radiology practitioners and interns showed no statistically significant differences with respect to other factors, including sex, age, country where their degree was obtained, marital status, specialty, professional rank, and years of experience.

**Table 3:** Comparative mean GAD-7 scores for participants with anxiety symptoms.

Variable		Anxiety (GAD-7 ≥ 8)		Overall Mean ± SD	P-value	Post-hoc
		n	%			
**Total (n = 230)**		98	42.6	11.07 ± 3.2		
**Gender**	Male (n = 153)	58	25.2	10.98 ± 3.1	0.545^b^	
	Female (n = 77)	40	17.4	11.20 ± 3.4		
**Age**	21 - 30 (n = 122)	46	20	10.02 ± 2.4	0.846^a^	
	31 - 40 (n = 64)	35	15.2	12.34 ± 3.6		
	41 - 60 (n = 44)	17	7.4	11.29 ± 3.33		
**Nationality**	Saudi Nationality (n =225)	98	42.6	11.07 ± 3.2	–	
	Other Nationality (n = 5)	0	0	0		
**Country of Radiologic Technology degree**	Saudi Arabia (n = 208)	92	40.0	10.96 ± 3.1	0.166^b^	
	Other (n = 22)	6	2.6	12.66 ± 4.13		
**Marital Status**	Single (n = 120)	49	21.3	10.81 ± 3.01	0.274^a^	
	Married (n = 102)	45	19.6	11.48 ± 3.44		
	Divorced (n = 8)	4	1.7	9.50 ± 1.3		
**Healthcare Institution**	KAMC-Jeddah (n =72)	28	12.2	10.07 ± 2.8	0.035^*a^	KAMC-Riyadh VS KAMC-Jeddah (0.015), KAMC-Al Ahsa (0.001), KAHOC (0.034)
	KAMC-Riyadh (n = 22)	4	1.7	15 ± 4.96		
	KAMC-Al Ahsa (n = 40)	12	5.2	9.83 ± 1.46		
	KAHOC (n = 15)	4	1.7	11.25 ± 3.8		
	KSMC (n = 81)	50	21.8	11.60 ± 3.2		
**Medical Imaging Division**	Radiography/Mammography/Fluoroscopy (n = 98)	50	21.7	10.78 ± 7.47	0.926^a^	
	Magnetic Resonance Imaging (MRI) (n = 22)	9	3.9	10.88 ± 7.13		
	Computed Tomography (CT) (n = 49)	17	7.4	11.29 ± 3.86		
	Nuclear Medicine (n = 16)	4	1.7	11.25 ± 1.70		
	Ultrasonography (n = 25)	10	4.4	11.50 ± 2.79		
	Angiography (n = 20)	8	3.5	12 ± 2.07		
**Professional Rank**	Technician (n = 20)	9	3.9	12.33 ± 3.67	0.641^a^	
	Technologist/Specialist (n = 147)	68	29.6	10.73 ± 2.88		
	Senior Specialist (n = 36)	11	4.8	12.36 ± 4.84		
	Consultant (n = 4)	1	0.4	11 ± 0		
	Intern (n = 23)	9	3.9	10.77 ± 2.27		
**Years of Experience**	Less than 1 year (n = 55)	21	9.1	11 ± 3	0.462^a^	
	1- 3 years (n = 35)	14	6.1	9.21 ± 1.47		
	4 - 5 years (n = 36)	18	7.8	9.77 ± 1.59		
	6 + years (n = 104)	45	19.6	12.20 ± 3.68		

- KAMC, King Abdulaziz Medical City.

- KSMC, King Saud Medical City.

- KAHOC, King Abdulaziz Hospital and Oncology Center.

- a, Kruskal Wallis test.

- b = Mann-Whitney test.

- * = P < 0.05 (i.e., Significance).

### 3.4 Average scores of participants on MBI-HSS (MP) burnout dimensions and items

**Table 4** presents the comparative mean scores of the burnout dimensions among male and female radiology practitioners and interns. The mean scores of radiology practitioners and interns did not show a statistically significant difference, either overall or for each individual item constituting the EE dimension. For the DP scale items 10 and 11, which assessed fear of becoming emotionally hardened and exhibiting greater callousness towards others since starting work, female radiology practitioners and interns recorded significantly higher mean scores than their male counterparts, with p-values of 0.024 and 0.005, respectively. This finding suggests higher levels of burnout among females. Conversely, the average score for Item 19 on the PA scale, which reflects achieving rewarding work objectives, was significantly lower for male radiology practitioners and interns than for females (p = 0.019), suggesting higher burnout levels among males. Additionally, the average score for PA Item 21, which assesses ease in handling emotional problems at work, was significantly lower for females than for males (p = 0.009), further indicating higher burnout levels in female radiology practitioners and interns.

**Table 4:** Comparative mean scores of burnout dimensions among radiology practitioners and interns.

MBI-HSS-MP Subscale/Item		Burnout level (Mean ± SD)			P-value Male vs Female
		Total (n = 230)	Male (n = 153)	Female (n = 77)	
**Emotional Exhaustion (EE)**	SUM method	28.59 ± 11.26	28.44 ± 11.86	28.87 ± 10.04	0.556
	AVE method	3.18 ± 1.25	3.16 ± 1.32	3.21 ± 1.12	0.556
Item 1		3.33 ± 1.66	3.33 ± 1.68	3.31 ± 1.63	0.939
Item 2		3.5 ± 1.74	3.48 ± 1.78	3.55 ± 1.67	0.664
Item 3		3.2 ± 1.77	3.12 ± 1.81	3.38 ± 1.69	0.167
Item 6		3.43 ± 1.84	3.42 ± 1.91	3.45 ± 1.69	0.564
Item 8		3.35 ± 1.82	3.35 ± 1.91	3.35 ± 1.65	0.715
Item 13		3.27 ± 1.78	3.27 ± 1.81	3.26 ± 1.72	0.92
Item 14		3.06 ± 1.67	3.01 ± 1.72	3.16 ± 1.59	0.27
Item 16		2.96 ± 1.75	3 ± 1.81	2.87 ± 1.62	0.948
Item 20		2.49 ± 1.39	2.46 ± 1.44	2.55 ± 1.28	0.311
**Depersonalization (DP)**	SUM method	13.88 ± 5.84	13.55 ± 5.9	14.53 ± 5.71	0.235
	AVE method	2.78 ± 1.17	2.71 ± 1.18	2.91 ± 1.14	0.235
Item 5		2.26 ± 1.34	2.26 ± 1.39	2.26 ± 1.22	0.492
Item 10		2.71 ± 1.6	2.58 ± 1.62	2.97 ± 1.56	0.024*
Item 11		2.83 ± 1.65	2.65 ± 1.64	3.17 ± 1.63	0.005*
Item 15		2.6 ± 1.48	2.56 ± 1.5	2.69 ± 1.46	0.329
Item 22		3.48 ± 1.81	3.5 ± 1.85	3.44 ± 1.74	0.842
**Personal Accomplishment (PA)**	SUM method	38.33 ± 11.59	38.11 ± 11.8	38.75 ± 11.23	0.761
	AVE method	4.79 ± 1.45	4.76 ± 1.47	4.84 ± 1.4	0.761
Item 4		4.9 ± 2.04	4.9 ± 2.03	4.92 ± 2.08	0.659
Item 7		5.06 ± 2.03	5.07 ± 2.07	5.04 ± 1.96	0.707
Item 9		4.75 ± 1.91	4.72 ± 1.85	4.81 ± 2.03	0.696
Item 12		4.37 ± 1.82	4.2 ± 1.86	4.69 ± 1.7	0.062
Item 17		4.74 ± 1.98	4.65 ± 2.03	4.92 ± 1.88	0.36
Item 18		4.65 ± 1.94	4.68 ± 1.99	4.58 ± 1.85	0.654
Item 19		5.17 ± 1.84	4.97 ± 1.87	5.55 ± 1.72	0.019*
Item 21		4.7 ± 1.93	4.92 ± 1.95	4.25 ± 1.84	0.009*

- * = P < 0.05 (i.e., significance).

### 3.5 Prevalence and variability of burnout levels, stratified according to study variables

**Figure 2:**
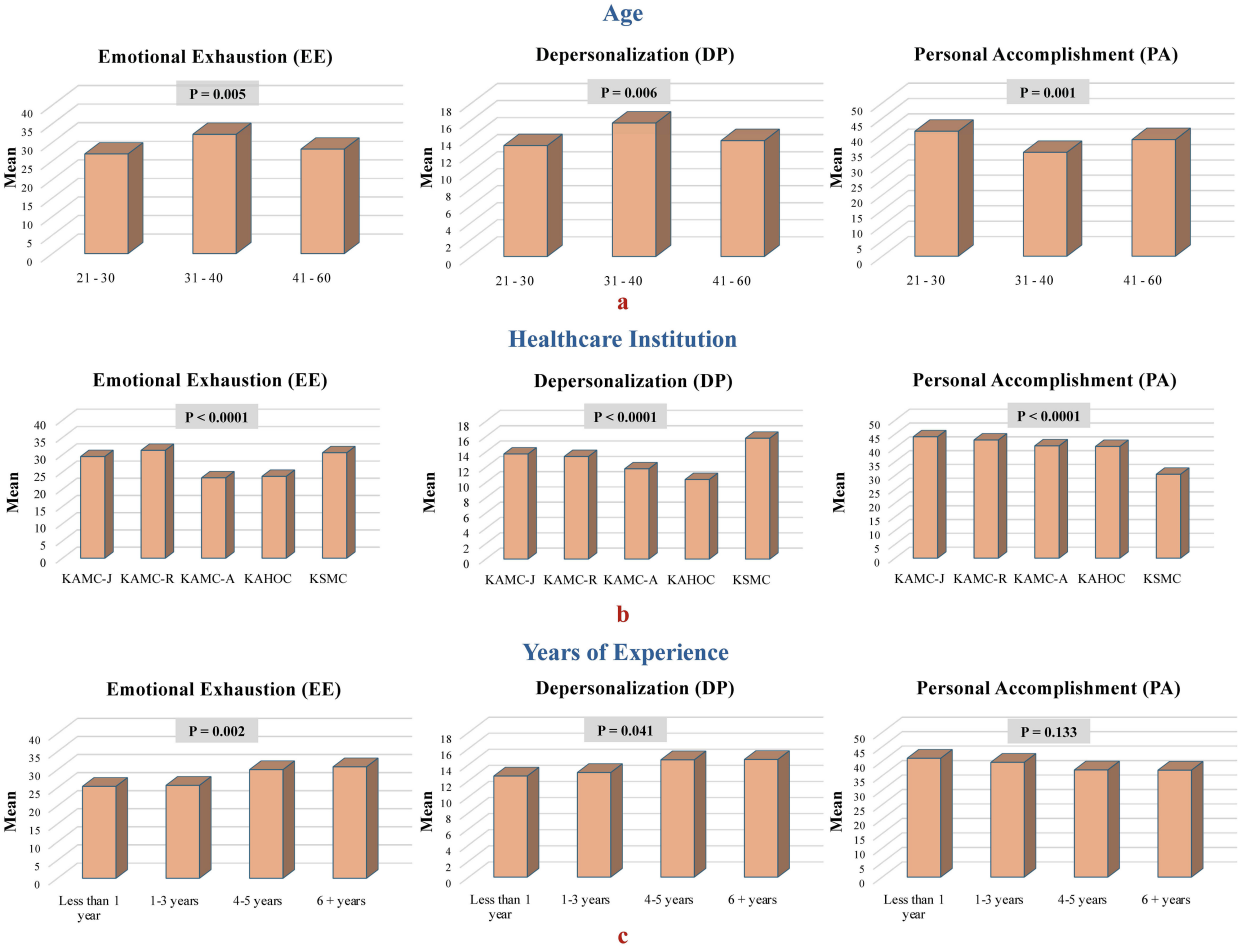
Comparison of mean burnout dimension scores among participants, categorized by **(A)** age, **(B)** healthcare institution, and **(C)** years of experience.

**Table 5** presents a descriptive analysis detailing the incidence and variability of burnout among the participants. These are categorized into three distinct burnout categories organized according to various study variables. **Table 6**, on the other hand, provides inferential analysis, highlighting comparisons made within the study variables. Within the entire sample, the proportions of radiology practitioners and interns at moderate to high risk of burnout in the EE, DP, and PA dimensions were 82.3%, 93.5%, and 52.1%, respectively. Differences in burnout levels among radiology practitioners and interns are associated with various factors, including age and nationality, particularly for the PA dimension. Additionally, the institution of affiliation, specialty, and professional rank influence PA, while years of service are associated with EE and DP dimensions of burnout. Individuals aged 31–40 years demonstrated significantly elevated burnout levels compared with their younger counterparts, with statistical significance observed in the EE (p = 0.008), DP (p = 0.008), and PA (p = 0.001) dimensions. Although burnout levels were higher in this age group than in the other older age groups, the differences were not statistically significant (**Figure 2A**). Furthermore, individuals affiliated with the KSMC exhibited significantly elevated burnout levels than those from other institutions (p < 0.0001), spanning the EE, DP, and PA dimensions of burnout (**Figure 2B**). Radiology practitioners and interns in MRI and angiography showed significantly lower average scores on the PA scale than those in radiography/mammography/fluoroscopy (p = 0.011), indicating higher levels of burnout. However, although lower, this difference did not reach statistical significance when compared with other specializations. Conversely, radiology interns exhibited significantly higher scores on the PA scale than technologists (p = 0.015), suggesting lower levels of burnout among interns. However, despite these higher scores, the difference was not statistically significant when compared with other radiology professionals. Moreover, radiology practitioners and interns possessing four or more years of experience demonstrated markedly higher mean scores on the EE (p = 0.002) and DP scales (p = 0.041) than their counterparts with fewer years of experience, suggesting increased levels of burnout (**Figure 2C**). Burnout levels among radiology practitioners and interns showed no statistically significant correlation with other factors, including sex, the country where their degree was obtained, and marital status.

**Table 5:** Prevalence of burnout among participants: descriptive analyses.

Variable	MBI-HSS-MP Subscale														
	Emotional Exhaustion (EE)					Depersonalization (DP)					Personal Accomplishment (PA)				
	SUM Method	AVE Method	Low	Moderate	High	SUM Method	AVE Method	Low	Moderate	High	SUM Method	AVE Method	Low	Moderate	High
	Mean ± SD		Mean ± SD; n (%)			Mean ± SD		Mean ± SD; n (%)			Mean ± SD		Mean ± SD; n (%)		
**Total**	28.59 ± 11.260	3.17± 1.251	14.7 ± 2.9; 41 (17.8)	22.6 ± 2.2; 69 (30)	36.8 ± 9.2; 119 (52.2)	13.88 ± 5.841	2.77 ± 1.168	5 ± 0; 15 (6.5)	7.5 ± 1.3; 30 (13)	15.6 ± 5.1; 185 (80)	38.33 ± 11.591	4.79 ± 1.448	48.5 ± 5.1; 112 (48.7)	36.7 ± 1.8; 35 (15.2)	25.3 ± 4.9; 83 (36.1)
Gender															
Male (n = 153)	28.44 ± 11.857	3.16 ± 1.3175	13.9 ± 2.9; 30 (13)	22.6 ± 2.2; 47 (20.4)	37.8 ± 9.2; 76 (33)	13.55 ± 5.902	2.71 ± 1.1803	5 ± 0; 12 (5.2)	7.2 ± 1.2; 23 (10)	15.7 ± 5; 118 (51.3)	38.11 ± 11.797	4.764 ± 1.4747	48.5 ± 5.2; 75 (32.6)	36.7 ± 1.8; 19 (8.3)	25.4 ± 5; 59 (25.7)
Female (n = 77)	28.87 ± 10.037	3.208 ± 1.1152	16.9 ± 1.6; 11 (4.8)	22.6 ± 2.5; 22 (9.6)	35 ± 8.9; 44 (19.1)	14.53 ± 5.705	2.906 ± 1.1409	5 ± 0; 3 (1.3)	8.4 ± 1.1; 7 (3)	15.6 ± 5.3; 67 (29.1)	38.75 ± 11.232	4.844 ± 1.404	48.5 ± 4.9; 37 (16.1)	36.8 ± 1.7; 16 (7)	25 ± 4.8; 24 (10.4)
Age															
21 - 30 (n = 122)	26.88 ± 10.228	2.986 ± 1.1364	15.3 ± 2.7; 26 (6.1)	21.9 ± 2.2; 40 (13.3)	35.8 ± 7.9; 56 (30.5)	13.03 ± 5.485	2.607 ± 1.0969	5 ± 0; 8 (1.3)	7.5 ± 1.2; 19 (4.5)	14.8 ± 4.9; 95 (44.1)	40.75 ± 10.86	5.093 ± 1.3575	48.1 ± 4.8; 73 (39.8)	37.5 ± 1.5; 19 (8.1)	24.9 ± 5.7; 30 (8.5)
31 - 40 (n = 64)	32.13 ± 11.798	3.569 ± 1.3109	13.9 ± 3.2; 7 (1.5)	23.5 ± 2.2; 12 (4.3)	37.3 ± 9.8; 45 (25.5)	15.67 ± 5.787	3.134 ± 1.1575	5 ± 0; 3 (0.5)	7.8 ± 1.6; 5 (1.2)	16.9 ± 5; 56 (29.7)	33.91 ± 10.584	4.238 ± 1.323	48.7 ± 4.9; 18 (9.9)	35.9 ± 1.9; 10 (4.1)	26 ± 3.4; 36 (10.6)
41 - 60 (n = 44)	28.18 ± 12.242	3.131 ± 1.3603	13.5 ± 3.1; 8 (1.6)	23.6 ± 1.7; 17 (6.1)	38.4 ± 11.2; 19 (11.1)	13.61 ± 6.417	2.723 ± 1.2833	5 ± 0; 4 (0.6)	7 ± 1.3; 6 (1.3)	15.7 ± 5.6; 34 (16.8)	38.05 ± 13.178	4.756 ± 1.6473	49.8 ± 6.08; 21 (11.9)	35.7 ± 1.2; 6 (2.4)	24.4 ± 6.1; 17 (4.7)
Nationality															
Saudi Nationality (n =225)	28.72 ± 11.247	3.191 ± 1.2497	14.7 ± 3; 39 (17)	22.6 ± 2.2; 66 (28.7)	36.7 ± 9.2; 119 (51.7)	13.96 ± 5.788	2.793 ± 1.1576	5 ± 0; 13 (5.7)	7.5 ± 1.2; 29 (12.6)	15.6 ± 5.1; 182 (79.1)	38.07 ± 11.564	4.759 ± 1.4455	48.4 ± 5.1; 107 (46.5)	36.8 ± 1.8; 34 (14.8)	25.3 ± 4.9; 83 (36.1)
Other Nationality (n = 5)	22.6 ± 11.349	2.511 ± 1.261	14.5 ± 0.7; 2 (0.9)	21 ± 1.4; 2 (0.9)	42 ± 0; 1 (0.4)	10 ± 7.681	2 ± 1.5362	5 ± 0; 2 (0.9)	0 ± 0, 0(0)	17 ± 8.5; 2 (0.9)	49.8 ± 5.848	6.225 ± 0.731	49.8 ± 5.8; 5 (2.2)	0 ± 0, 0(0)	0 ± 0, 0(0)
Country of Radiologic Technology degree															
Saudi Arabia (n = 208)	28.61 ± 11.265	3.179 ± 1.2516	14.6 ± 2.9; 39 (17)	22.6 ± 2.2; 62 (27)	36.7 ± 9; 106 (46.1)	13.94 ± 5.798	2.788 ± 1.1597	5 ± 0; 14 (6.1)	7.7 ± 1.2; 26 (11.3)	15.5 ± 5.1; 167 (72.6)	38.17 ± 11.269	4.772 ± 1.4086	48.4 ± 5.1; 100 (43.5)	36.9 ± 1.7; 31 (13.5)	25.1 ± 4.9; 76 (33)
Other (n = 22)	28.36 ± 11.479	3.152 ± 1.2754	17 ± 0; 2 (0.9)	22.6 ± 2.6; 7 (3)	37.3 ± 11.1; 13 (5.7)	13.27 ± 6.356	2.655 ± 1.2712	5 ± 0; 1 (0.4)	6.3 ± 0.5; 4 (1.7)	16.8 ± 5.1; 17 (7.4)	39.77 ± 14.517	4.972 ± 1.8147	49.3 ± 5.1; 12 (5.2)	35.8 ± 2.4; 4 (1.7)	27.5 ± 4.4; 6 (2.6)
Marital status															
Single (n = 120)	27.5 ± 10.803	3.056 ± 1.2003	15 ± 2.7; 23 (10)	22.3 ± 2.3; 40 (17.4)	36.8 ± 9.2; 57 (24.8)	13.63 ± 5.938	2.727 ± 1.1876	5 ± 0; 10 (4.3)	7.8 ± 1.2; 16 (7)	15.4 ± 5.7; 94 (40.9)	39.84 ± 11.202	4.98 ± 1.4003	48.4 ± 4.9; 65 (28.3)	36.8 ± 1.7; 24 (10.4)	24.3 ± 5.3; 31 (13.5)
Married (n = 102)	29.91 ± 11.815	3.324 ± 1.3127	14.3 ± 0; 15 (6.5)	23 ± 2.1; 26 (11.3)	36.5 ± 9.1; 60 (26.1)	14.15 ± 5.871	2.829 ± 1.1741	5 ± 0; 4 (1.7)	7.1 ± 1.3; 14 (6.1)	16 ± 4.5; 83 (36.1)	36.68 ± 12.044	4.585 ± 1.5055	48.4 ± 0; 45 (19.6)	36.8 ± 2.1; 9 (3.9)	25.9 ± 4.7; 47 (20.4)
Divorced (n = 8)	28 ± 10.184	3.111 ± 1.1316	15 ± 2.6; 3 (1.3)	23.3 ± 3.8; 3 (1.3)	47.5 ± 10.6; 2 (0.9)	14.13 ± 4.224	2.825 ± 0.8447	5 ± 0; 1 (0.4)	0 ± 0, 0(0)	14.1 ± 4.1; 7 (3)	36.63 ± 9.288	4.578 ± 1.161	54 ± 0; 2 (0.9)	36.5 ± 2.1; 2 (0.9)	26 ± 4.2; 4 (1.7)
Healthcare Institution															
KAMC-Jeddah (n =72)	29.47 ± 9.361	3.275 ± 1.0401	16.2 ± 2.3; 10 (4.3)	22.2 ± 2.5; 25 (10.9)	36.2 ± 7.9; 37 (16.1)	13.74 ± 5.289	2.747 ± 1.0578	5 ± 0; 4 (1.7)	8 ± 1; 3 (1.3)	14.4 ± 5; 65 (28.3)	44.03 ± 8.049	5.503 ± 1.0061	48.1 ± 4.9; 49 (21.3)	37.1 ± 1.9; 15 (6.5)	27.3 ± 7.5; 8 (3.5)
KAMC-Riyadh (n = 22)	31.27 ± 15.097	3.475 ± 1.6775	13 ± 5.7; 2 (0.9)	23.8 ± 1.5; 10 (4.3)	37.7 ± 11.9; 9 (3.9)	13.41 ± 6.573	2.682 ± 1.3146	5 ± 0; 1 (0.4)	6.3 ± 0.5; 4 (1.7)	14.7 ± 4; 16 (7)	42.86 ± 12.594	5.358 ± 1.5742	49.2 ± 0; 15 (6.5)	0 ± 0, 0(0)	23.7 ± 8; 6 (2.6)
KAMC-Al Ahsa (n = 40)	23.3 ± 9.544	2.589 ± 1.0604	14.7 ± 2.8; 15 (6.5)	21.4 ± 1; 12 (5.2)	36.6 ± 10.5; 13 (5.7)	11.8 ± 5.827	2.36 ± 1.1655	5 ± 0; 6 (2.6)	7.3 ± 1.5; 10 (4.3)	14.5 ± 5.5; 24 (10.4)	40.73 ± 12.488	5.091 ± 1.561	49.7 ± 0; 22 (9.6)	36.9 ± 1.9; 7 (3)	22.8 ± 5.5; 11 (4.8)
KAHOC (n = 15)	23.73 ± 17.023	2.637 ± 1.8914	13 ± 3.1; 5 (2.2)	24 ± 1.9; 3 (1.3)	42 ± 12.3; 7 (3)	10.4 ± 7.298	2.08 ± 1.4595	5 ± 0; 3 (1.3)	6.8 ± 1.2; 4 (1.7)	17.9 ± 5.8; 8 (3.5)	40.53 ± 14.116	5.067 ± 1.7645	49.5 ± 5.1; 10 (4.3)	36 ± 0; 1 (0.4)	26.8 ± 4.3; 4 (1.7)
KSMC (n = 81)	30.58 ± 10.253	3.398 ± 1.1392	14.6 ± 2.9; 9 (3.9)	23.1 ± 2.2; 19 (8.3)	36.4 ± 9; 53 (23)	15.8 ± 5.236	3.16 ± 1.0471	5 ± 0; 1 (0.4)	8.3 ± 1; 9 (3.9)	17.1 ± 4.9; 71 (30.9)	30.43 ± 8.528	3.804 ± 1.066	46.8 ± 5.7; 16 (7)	36.3 ± 1.6; 12 (5.2)	25.6 ± 3.8; 53 (23)
Specialty															
Radiography/ Mammography/ Fluoroscopy (n = 98)	29.45 ± 12.274	3.272 ± 1.3637	14.9 ± 3; 20 (8.7)	22.1 ± 2.3; 28 (12.2)	37 ± 9.2; 50 (21.7)	14.24 ± 6.28	2.849 ± 1.2561	5 ± 0; 9 (3.9)	6.9 ± 0.9; 11 (4.8)	15.7 ± 5.5; 78 (33.9)	40.86 ± 11.388	5.107 ± 1.4235	49.3 ± 5.3; 51 (22.2)	36.9 ± 1.8; 19 (8.3)	26.1 ± 4.6; 28 (12.2)
Magnetic Resonance Imaging (MRI) (n = 22)	27.5 ± 9.262	3.056 ± 1.0291	15.4 ± 2.4; 5 (2.2)	23.5 ± 3; 4 (1.7)	31.7 ± 6.1; 13 (5.7)	13.77 ± 5.681	2.755 ± 1.1363	0 ± 0, 0(0)	8 ± 1.4; 4 (1.7)	14.7 ± 4.6; 18 (7.8)	32.59 ± 10.349	4.074 ± 1.2937	45.7 ± 5.2; 11 (4.8)	37 ± 2.6; 3 (1.3)	24.3 ± 4.7; 8 (3.5)
Computed Tomography (CT) (n = 49)	28.49 ± 13.161	3.166 ± 1.4623	13.4 ± 3.4; 7 (3)	23.1 ± 1.5; 19 (8.3)	38.4 ± 10.2; 22 (9.6)	13.02 ± 6.306	2.604 ± 1.2613	5 ± 0; 3 (1.3)	7.9 ± 2.1; 7 (3)	15.3 ± 4.9; 38 (16.5)	38 ± 11.762	4.75 ± 1.4702	48.2 ± 4.4; 24 (10.4)	37.2 ± 1.5; 5 (2.2)	23.4 ± 6.5; 19 (8.3)
Nuclear Medicine (n = 16)	27.75 ± 8.82	3.083 ± 0.98	15.3 ± 1.5; 3 (1.3)	23.3 ± 2.3; 4 (1.7)	39.3 ± 12.5; 9 (3.9)	13.94 ± 5.17	2.788 ± 1.034	5 ± 0; 2 (0.9)	7.5 ± 1.3; 2 (0.9)	17.6 ± 6.8; 12 (5.2)	39.75 ± 11.091	4.969 ± 1.3863	48.2 ± 4.7; 5 (2.2)	35.6 ± 1.8; 5 (2.2)	25 ± 4.7; 6 (2.6)
Ultrasonography (n = 25)	27.36 ± 6.975	3.04 ± 0.775	13.7 ± 4.2; 3 (1.3)	21.7 ± 1.9; 9 (3.9)	35.8 ± 6; 13 (5.7)	13.88 ± 4.096	2.776 ± 0.8192	5 ± 0; 1 (0.4)	7.3 ± 1.2; 3 (1.3)	14.5 ± 4.6; 21 (9.1)	37.76 ± 11.069	4.72 ± 1.3836	47.9 ± 4.8; 14 (6.1)	38 ± 0; 1 (0.4)	27.1 ± 4.4; 10 (4.3)
Angiography (n = 20)	28 ± 9.782	3.111 ± 1.0869	16 ± 2; 3 (1.3)	24.2 ± 1.1; 5 (2.2)	37.7 ± 9.8; 12 (5.2)	14.25 ± 5.369	2.85 ± 1.0739	0 ± 0, 0(0)	8 ± 1.7; 3 (1.3)	17 ± 3.3; 17 (7.4)	32.6 ± 11.371	4.075 ± 1.4214	49.1 ± 5.7; 7 (3)	36 ± 1.4; 2 (0.9)	25.8 ± 2.4; 11 (4.8)
Professional rank															
Technician (n = 20)	28.95 ± 15.059	3.217 ± 1.6733	14.7 ± 1.8; 7 (3)	21.5 ± 0.7; 2 (0.9)	33.6 ± 6.7; 11 (4.8)	13.7 ± 7.774	2.74 ± 1.5548	5 ± 0; 1 (0.4)	7.4 ± 1.3; 5 (2.2)	14.3 ± 3.1; 14 (6.1)	37.85 ± 13.593	4.731 ± 1.6991	51.7 ± 5.1; 10 (4.3)	36.7 ± 2.3; 3 (1.3)	27.3 ± 3.7; 7 (3)
Technologist/ Specialist (n = 147)	28.61 ± 10.883	3.179 ± 1.2092	14.3 ± 3; 26 (11.3)	22.5 ± 2.3; 44 (19.1)	37.9 ± 9.6; 76 (33)	13.93 ± 5.78	2.785 ± 1.1561	5 ± 0; 13 (5.7)	7.8 ± 1.2; 20 (8.7)	16.2 ± 5.4; 113 (49.1)	37.71 ± 11.07	4.713 ± 1.3838	47.7 ± 4.9; 70 (30.4)	36.8 ± 1.8; 22 (9.6)	25.2 ± 4.8; 54 (23.5)
Senior Specialist (n = 36)	31.5 ± 12.143	3.5 ± 1.3492	15.5 ± 4.4; 4 (1.7)	23.8 ± 0; 11 (4.8)	34.8 ± 9.6; 21 (9.1)	14.53 ± 6.101	2.906 ± 1.2203	5 ± 0; 1 (0.4)	6.3 ± 0; 4 (1.7)	14.7 ± 4; 31 (13.5)	37.36 ± 12.053	4.67 ± 1.5066	48.1 ± 4.9; 19 (8.3)	35.3 ± 0; 4 (1.7)	24.3 ± 6.1; 13 (5.7)
Consultant (n = 4)	24.25 ± 4.787	2.694 ± 0.5319	0 ± 0, 0(0)	25 ± 1.8; 1 (0.4)	38.7 ± 11.1; 3 (1.3)	13.25 ± 2.217	2.65 ± 0.4435	0 ± 0, 0(0)	0 ± 0, 0(0)	16 ± 6.9; 4 (1.7)	30 ± 5.099	3.75 ± 0.6374	0 ± 0, 0(0)	0 ± 0, 0(0)	23 ± 2.4; 4 (1.7)
Intern (n = 23)	24.3 ± 7.98	2.7 ± 0.8866	17 ± 2; 4 (1.7)	21.9 ± 2.3; 11 (4.8)	35.5 ± 5; 8 (3.5)	12.83 ± 4.417	2.565 ± 0.8835	0 ± 0, 0(0)	6 ± 0; 1 (0.4)	14.7 ± 5.6; 22 (9.6)	45.65 ± 10.857	5.707 ± 1.3571	50.9 ± 5; 13 (5.7)	37.7 ± 1; 6 (2.6)	29 ± 4.5; 4 (1.7)
Years of experience															
Less than 1 year (n = 55)	25.38 ± 11.636	2.82 ± 1.2929	15.1 ± 2.6; 15 (6.5)	22.2 ± 2.4; 23 (10)	38.2 ± 9.3; 17 (7.4)	12.56 ± 6.03	2.513 ± 1.206	5 ± 0; 5 (2.2)	7.2 ± 1.3; 6 (2.6)	14.6 ± 5.1; 44 (19.1)	41.04 ± 12.235	5.13 ± 1.5294	49.8 ± 4.9; 30 (13)	37.4 ± 1.2; 12 (5.2)	23.8 ± 6.6; 13 (5.7)
1-3 years (n = 35)	25.66 ± 7.669	2.851 ± 0.8522	14.7 ± 3.3; 7 (3)	21.3 ± 2.1; 6 (2.6)	37.8 ± 11.2; 22 (9.6)	13 ± 5.156	2.6 ± 1.0313	5 ± 0; 3 (1.3)	7.9 ± 1; 8 (3.5)	16.3 ± 7.7; 24 (10.4)	39.63 ± 9.792	4.954 ± 1.224	47.8 ± 5.1; 19 (8.3)	35.9 ± 1.7; 9 (3.9)	23.4 ± 5.5; 7 (3)
4 - 5 years (n = 36)	30 ± 8.495	3.333 ± 0.9439	12 ± 3; 3 (1.3)	23.5 ± 0; 12 (5.2)	38 ± 8.3; 21 (9.1)	14.58 ± 4.831	2.917 ± 0.9661	5 ± 0; 4 (1.7)	0 ± 0, 0(0)	16.3 ± 4.6; 32 (13.9)	37 ± 10.575	4.625 ± 1.3219	48.2 ± 4.4; 18 (7.8)	37 ± 2; 3 (1.3)	26.6 ± 4.4; 15 (6.5)
6 + years (n = 104)	30.78 ± 12.37	3.42 ± 1.3745	14.9 ± 3.1; 16 (7)	22.9 ± 2; 28 (12.2)	35.6 ± 8.7; 59 (25.7)	14.62 ± 6.18	2.925 ± 1.2361	5 ± 0; 3 (1.3)	7.4 ± 1.4; 16 (7)	15.7 ± 4.3; 84 (36.5)	36.91 ± 11.976	4.614 ± 1.497	48 ± 5.4; 45 (19.6)	36.6 ± 2.1; 11 (4.8)	25.6 ± 4.4; 47 (20.4)

- KAMC, King Abdulaziz Medical City.

- KSMC, King Saud Medical City.

- KAHOC, King Abdulaziz Hospital and Oncology Center.

- Emotional Exhaustion (Low: score <19, Moderate: score >=19-26>= score, High: score>26).

- Depersonalization (Low: score <6, Moderate: score >=6-9>= score, High: score>9).

- Personal Accomplishment (Low: score>39, Moderate: score >=34-39>= score, High: score <34).

**Table 6:** Prevalence of burnout among participants: inferential analyses. .

Variable		MBI-HSS-MP Subscale					
		Emotional Exhaustion (EE)		Depersonalization (DP)		Personal Accomplishment (PA)	
		P-value	Post-hoc	P-value	Post-hoc	P-value	Post-hoc
**Gender**	Male	0.556^b^	**-**	0.235^b^	**-**	0.761^b^	**-**
	Female						
**Age**	21 - 30	0.005^*a^	20 - 30 vs 31 - 40 (0.004)	0.006^*a^	20 - 30 vs 31 - 40 (0.004)	0.001^*a^	20 - 30 vs 31 - 40 (0.000)
	31 - 40						
	41 - 60						
**Nationality**	Saudi Nationality	0.114^b^	**-**	0.109^b^	**-**	0.021^*b^	**-**
	Other Nationality						
**Country of Radiologic Technology degree**	Saudi Arabia	0.962^b^	**-**	0.658^b^	**-**	0.359^b^	**-**
	Other						
**Marital Status**	Single	0.226^a^	**-**	0.707^a^	**-**	0.123^a^	**-**
	Married						
	Divorced						
**Healthcare Institution**	KAMC-Jeddah	<0.0001*a	KAHOC vs KSMC (0.016); KAMC-Al Ahsa vs KAMC-Jeddah (0.004); KAMC-Al Ahsa vs KSMC (<0.0001)	<0.0001*a	KAHOC vs KSMC (0.002); KAMC-Al Ahsa vs KSMC (<0.0001)	<0.0001*a	KAMC-Al Ahsa vs KSMC (<0.0001) KAHOC vs KSMC (0.010); KSMC vs KAMC-Riyadh (<0.0001); KSMC vs KAMC-Jeddah (<0.0001)
	KAMC-Riyadh						
	KAMC-Al Ahsa						
	KAHOC						
	KSMC						
**Medical Imaging Division**	Radiography/Mammography/ Fluoroscopy	0.901^a^		0.804^a^		0.011^*a^	Radiography/Mammography/Fluoroscopy vs MRI (0.038)
	Magnetic Resonance Imaging (MRI)						
	Computed Tomography (CT)						
	Nuclear Medicine						
	Ultrasonography						
	Angiography						
**Professional Rank**	Technician	0.153^a^		0.888^a^		0.012^*a^	Technologist/Specialist vs Intern (0.015)
	Technologist/Specialist						
	Senior Specialist						
	Consultant						
	Intern						
**Years of Experience**	Less than 1 year	0.002^*a^	Less than 1 year vs 6 + years (0.008) Less than 1 year vs 4 - 5 years (0.019)	0.041^*a^	Less than 1 year vs 6 + years (0.0013)	0.133^a^	
	1-3 years						
	4 - 5 years						
	6 + years						

- KAMC, King Abdulaziz Medical City.

- KSMC, King Saud Medical City.

- KAHOC, King Abdulaziz Hospital and Oncology Center.

- a = Kruskal Wallis test.

- b = Mann-Whitney test.

- * = P < 0.05 (i.e., Significance).

## 4 Discussion

This cross-sectional study explored the psychological well-being of radiology practitioners and interns across multiple tertiary care centers in various regions of Saudi Arabia. This study highlighted several key findings: First, 42.6% of radiology practitioners and interns reported experiencing GAD. Second, a substantial proportion of this group was identified as being at a moderate to high risk of burnout—82.3% for EE, 93.5% for DP, and 52.1% for PA. Furthermore, the mean EE and DP scores were notably higher among radiology practitioners and interns than the MBI-HSS norms for both the medical field and other occupational groups, whereas the PA scores were comparable (80). Additionally, female radiology practitioners and interns displayed a greater propensity for burnout, particularly when dealing with emotional challenges at work, and showed increased indifference towards others. Finally, those aged 31–40 years exhibited higher levels of burnout than both younger and older colleagues.

The mental health challenges faced by HCWs in Saudi Arabia during the COVID-19 pandemic have highlighted a significant issue mirroring global trends in the medical field. Heightened stress and anxiety were fueled by the fear of contracting the virus, concerns about transmitting it to loved ones, and the pressures of caring for numerous COVID-19 patients (85). In a study involving 4,920 HCWs in Saudi Arabia during the peak of the 2020 COVID-19 pandemic, anxiety levels were categorized as low (31.5%), moderate (36.1%), or high (32.3%). Several factors were identified as contributing to elevated anxiety levels, including being unmarried, working in nursing, radiology, or respiratory therapy, and cohabiting with the elderly, individuals with chronic illnesses, immune deficiencies, or respiratory conditions. Organizational factors, notably those working in settings with COVID-19 patients, were also significant (56). Among the 193 radiology workers in this sample, 26.4% reported low anxiety, 38.3% moderate anxiety, and 35.2% high anxiety. Our study was conducted between October and November 2023, following the WHO declaration on May 5, 2023, ending the global PHE for COVID-19, over three years after the pandemic began (74). This revealed a reduction in anxiety levels among radiology practitioners and interns, with medium and high anxiety levels decreasing by 18.3% and 6.5%, respectively. This decline in anxiety levels further highlights the significant impact of the COVID-19 peak period on the mental health of radiology professionals, especially compared with the post-pandemic era.

In this study, we explored the relationship between sex, age, and institutional affiliation and their impact on anxiety levels. Although not statistically significant, female participants reported higher anxiety levels than male participants. Furthermore, individuals aged 31–40 years exhibited the highest anxiety levels compared with both younger and older cohorts. These findings align with those of numerous recent investigations on HCWs in Saudi Arabia (54–57, 86–89). For instance, a 2022 cross-sectional analysis of 326 HCWs in Saudi Arabia indicated that female HCWs experienced greater anxiety than their male counterparts. Additionally, those in the 30–39 age group showed higher anxiety levels than individuals in other age groups, with variations in anxiety levels according to the type of healthcare facility (50). Another study conducted in March 2020 with 502 HCWs in Saudi Arabia found that female participants had higher average anxiety scores than males. In particular, those in the 30–39 age group reported higher anxiety levels than those in the other age groups (86). This increased anxiety in women under 39 years of age may be partly due to the dual burden of professional responsibilities and childcare, especially during lockdowns when school-aged children could not attend school. Concerns about contracting and transmitting the virus to children may further exacerbate anxiety levels among females with children (50, 86).

Previous research has identified a significant link between HCWs’ nationality and anxiety development (54, 57, 89). Nevertheless, the results of our study are not directly comparable because of the disproportionate composition of our sample, which predominantly included Saudi radiology practitioners and interns (97.8%), with minimal representation of non-Saudis (2.2%). This discrepancy also extends to the origin of their Radiological Technologist degrees; 90.4% of the participants obtained their degrees from Saudi Arabia, while only 9.6% received their degrees from other countries. In the present study, no correlation was observed between anxiety and marital status. This finding contrasts with a previous study conducted in Saudi Arabia during the peak of the COVID-19 pandemic in 2020, which indicated that married HCWs reported lower anxiety levels (56). This outcome is somewhat paradoxical, as it was initially hypothesized that concerns about transmitting the virus to family members would lead to increased stress during the pandemic.

We found no significant variations in anxiety levels based on radiology practitioners’ and interns’ years of professional experience or professional standing. This finding contrasts with the mixed results of previous studies on the relationship between HCWs’ experiences and anxiety symptoms. Some studies identified a noteworthy link between higher levels of experience and anxiety symptoms among HCWs (54, 89, 90). For instance, one study highlighted that a substantial proportion of HCWs with over 10 years of experience exhibited moderate to severe anxiety (54). Similarly, a study from Turkey noted that participants with a decade or more of service had significantly elevated depression and anxiety scores compared with their counterparts with less than 10 years of experience (90). Conversely, research involving 389 HCWs from government and private hospitals in Saudi Arabia indicated that those with less experience were more prone to stress and anxiety (55). High anxiety levels were particularly associated with HCWs with less than 5 years of experience (56). Additionally, a systematic review encompassing 55 studies from 2002 to August 21, 2020, observed that increasing professional experience correlated with a decreased likelihood of HCWs developing psychiatric disorders (91). Moreover, extensive professional experience has been suggested as a potential protective factor against psychopathological distress in HCWs (92).

Burnout has been a major issue for HCWs in Saudi Arabia during the COVID-19 pandemic, driven by prolonged working hours, increased workloads, and the emotional burden of patient care (93). The prevalence of burnout among HCWs in Saudi Arabia varies significantly, with the majority of studies relying on cross-sectional designs and convenience sampling techniques. Specifically, there is a lack of comparative data among radiology practitioners. Our search yielded only a single relevant study conducted in Saudi Arabia prior to the COVID-19 pandemic, involving 150 radiographers (44). Using the MBI-HSS as a measurement tool, this study found that 67% of the radiographers faced a moderate-to-high risk of burnout in terms of EE, 52% in DP, and 58% in PA. Additionally, the study recorded mean scores (with standard deviation) for EE, DP, and PA as 21.44 (± 13.0), 8.12 (± 6.99), and 35.63 (± 8.59), respectively. In comparison, our study revealed a significantly higher incidence of burnout, particularly in the domains of EE (82.3%) and DP (93.5%), along with increased mean scores for EE (28.59 ± 11.3) and DP (13.9 ± 5.8). This indicates a more pronounced level of burnout among radiology practitioners in our sample compared with the findings of a prior study. The heightened occurrence and level of burnout among radiology practitioners in our study could reflect the lasting impact of the COVID-19 pandemic, even after its peak. The heightened incidence and severity of burnout may be attributed to specific factors during the COVID-19 pandemic in Saudi Arabia. The surge in COVID-19 cases has increased workload, leading to longer hours of stress (94). Changes in work conditions, including strict infection control measures, PPE usage, and hospital reorganization, added physical and mental strain (95). Additionally, increased anxiety and fear of infection, along with uncertainty and rapid changes in protocols, exacerbate stress. Studies have shown high psychological distress among healthcare workers in Saudi Arabia during the pandemic, which contributed to higher burnout rates (94, 95).

Several studies have revealed that moderate to high burnout rates among HCWs, particularly nurses, are associated with younger age, single status, nationality, extended working hours, and heavy workloads​​ (96). Another study emphasized acute levels of job burnout, especially prevalent among individuals in high-stress areas, such as emergency and intensive care units. This highlights the role of factors such as an overwhelming workload, intense time constraints, and the demanding nature of work in healthcare settings, in exacerbating burnout among HCWs ​ (97). In our study, we found no link between sex and either the occurrence or level of burnout. Furthermore, our findings revealed that individuals aged 31–40 years experienced the highest burnout levels across all dimensions compared with both younger and older groups. Similarly, a study of 646 HCWs in Saudi Arabia found no correlation between gender and burnout levels but identified the highest burnout prevalence among individuals aged 27–31 years (97). Another study of 239 HCWs in Saudi Arabia reported that female participants had higher levels of emotional exhaustion and depersonalization, with the highest burnout among those aged 23–28 (59). One theory suggests that younger individuals who are more engaged in social media experience higher stress levels due to information overload (97). Conversely, older individuals may manage stress better because of their greater knowledge and understanding of the pandemic (98).

A 2010 study involving 198 nurses in Saudi Arabia found that married nurses were more susceptible to emotional exhaustion than their unmarried counterparts (99). This contrasts with our findings, which demonstrated no correlation between marital status and burnout. Similarly, a recent study conducted during the COVID-19 pandemic among HCWs in Saudi Arabia found no association between marital status and burnout (97). These divergent results may emphasize the significant impact of COVID-19 on HCWs, highlighting that the pandemic’s toll was independent of marital status. Burnout levels among radiology practitioners and interns varied not only between different institutions but also across specialties/modalities within radiology departments. Such disparities may be linked to the distinct strategies implemented by these institutions and departments to combat burnout as well as the individual characteristics related to coping with the stressors associated with burnout. Our findings reveal that radiology practitioners and interns with three years of professional experience or less experience elevated levels of burnout, specifically emotional exhaustion and depersonalization. This observation is partially echoed in a recent study that reported a higher incidence of burnout among HCWs with 1-5 years of experience than among those with more than five years. These outcomes challenge the prevailing belief that extensive professional experience protects HCWs against the development of psychopathological distress in HCWs (92).

### 4.1 Limitations

The limitations of this study stem from the exclusion of certain etiological factors related to GAD and burnout. These factors encompass health and well-being, such as a history of COVID-19, comorbidities or chronic diseases, sleep quality, and smoking habits. Further research is warranted to explore the influence of sociodemographic characteristics, including the number of children and economic status, on these conditions. The use of non-probability sampling, a small population size, and the inclusion of the entire population without calculating a specific sample size pose limitations to the generalizability of our findings. Another limitation is that the scales used to measure GAD and burnout were not reviewed by psychologists or mental health experts, which may have affected our findings. Furthermore, our study exclusively examined GAD and did not explore other forms of anxiety disorders, such as Obsessive-Compulsive Disorder (OCD), social phobia, and panic disorder. Therefore, addressing these gaps in future research is essential.

### 4.2 Implication of the study

In response to our findings, immediate measures are essential to satisfy the psychological needs of vulnerable radiology practitioners and interns. Healthcare systems must develop and implement strategies to address this issue by incorporating immediate responses and long-term strategic planning. Monitoring the prevalence of GAD, burnout, and other psychological conditions remains imperative even after the post-PHE to ensure the well-being of healthcare professionals. Future research should investigate the impact of COVID-19 history, comorbidities, chronic diseases, sleep quality, smoking habits, and sociodemographic factors such as the number of children and economic status on GAD and burnout. A comprehensive approach that collectively examines these variables would provide a deeper understanding of their combined influence on GAD and burnout.

## 5 Conclusion

Our study indicated that 42.6% of radiology practitioners and interns reported experiencing GAD. Within this group, a significant number reported a heightened incidence of burnout, with 82.3% experiencing moderate-to-high levels of emotional exhaustion and 93.5% reporting moderate-to-high levels of depersonalization. Additionally, our research revealed a reduction in GAD levels among radiology practitioners and interns than those observed during the peak periods of the COVID-19 pandemic among radiology professionals. Furthermore, it revealed a markedly increased incidence and severity of burnout relative to the pre-pandemic data from a similar sample. This study highlights GAD and burnout as significant challenges for HCWs, including radiology practitioners and interns. Multiple factors contribute to the prevalence of GAD and burnout among HCWs. Stakeholders are urged to address these factors to alleviate their impact on HCWs in preparation for and response to pandemics or even post-pandemics. A comprehensive array of preventive strategies is crucial to mitigate GAD and burnout among radiology practitioners and interns. These strategies should include enhancing coping mechanisms, providing personalized support, boosting mental health awareness, and stressing the significance of supportive work environments. Specific programs aim to enhance coping mechanisms and raise mental health awareness among radiology practitioners and interns. Notable examples include the American College of Radiology’s Radiology Well-Being Program, which offers stress management workshops, peer support networks, access to specialized mental health professionals, and Employee Assistance Programs (EAPs) available in many hospitals that provide confidential counseling and support for personal or work-related stress. Additionally, ensuring manageable workloads and providing adequate resources and psychological support are imperative for individuals with challenging roles in the field of radiology.

## Data Availability

The raw data supporting the conclusions of this article will be made available by the authors, without undue reservation.
